# A biomechanical investigation of a novel intramedullary nail used to salvage failed internal fixations in intertrochanteric fractures

**DOI:** 10.1186/s13018-023-04112-w

**Published:** 2023-08-28

**Authors:** Ping Chen, Zhirong Fan, Nengneng Xu, Haizhou Wang

**Affiliations:** 1 The Second Affiliated Hospital of Guangzhou University of Traditional Chinese Medicine (Guangdong Provincial Hospital of Traditional Chinese Medicine), Guangzhou University of Chinese Medicine, Guangzhou, 510120 China; 2 Panyu Hospital of Chinese Medicine, Guangzhou University of Chinese Medicine, Guangzhou, 511401 China

**Keywords:** Intertrochanteric fracture, Revision surgery, PFBN, PFNA, FEA

## Abstract

**Purpose:**

The ideal approach for revision surgery following femoral head salvage treatments for an intertrochanteric fracture is still up for debate. A novel variety of proximal femoral bionic intramedullary nail (PFBN) has been created in clinical practice. We aimed to compare the biomechanical results of the novel implant to conventional intramedullary and extramedullary fixation in the treatment of intertrochanteric fracture following primary internal fixation failure.

**Methods:**

Using finite element analysis, we created a three-dimensional model of the intertrochanteric fracture's helical blade cut-out for this investigation. The PFBN 1 group, the PFBN 2 group, the PFNA group, and the DHS group were our four test groups. For each fracture group, the von Mises stress and displacements of the femur and internal fixation components were measured under 2100 N axial loads.

**Results:**

The values for the femoral displacement in the PFBN1 group, PFBN2 group, PFNA group, and DHS group were 6.802 mm, 6.716 mm, 8.080 mm, and 8.679 mm, respectively. The internal implant displacement values were 6.201 mm, 6.138 mm, 7.396 mm, and 8.075 mm in the PFBN1 group, PFBN2 group, PFNA group, and DHS group, respectively. The maximum von Mises Stress in the femoral was 187.2 MPa, 85.18 MPa, 106.6 MPa, and 386.2 MPa in the PFBN1 groups, PFBN2 groups, PFNA groups, and DHS groups, respectively. In the PFBN1 groups, PFBN2 groups, PFNA groups, and DHS groups, the maximum von Mises Stress in internal fixation was 586.7 MPa, 559.8 MPa, 370.7 MPa, and 928.4.8 MPa, respectively.

**Conclusion:**

Our biomechanical research demonstrates that intramedullary fixation is more stable than extramedullary fixation when salvaging failed internal fixations in intertrochanteric fracture. Compared with PFNA and DHS, PFBN showed better biomechanical stability in the treatment of patients with revised intertrochanteric fractures. In light of this, we advocate PFBN fixation as the method of choice for intertrochanteric fracture revision. This result still has to be confirmed in more clinical research.

## Introduction

Hip fractures are a frequent occurrence in elderly osteoporotic patients, who also have a high morbidity and mortality rate. Hip fractures are thought to occur in about 1.6 million people worldwide each year, and by the year 2050, that number is expected to rise to 4.5 million [1, 2]. Among all hip fractures, intertrochanteric fractures make up 41.5–50% of cases [3].

Early firm internal fixation is advised for patients with intertrochanteric fractures in order to facilitate early rehabilitation and minimize the negative effects of prolonged bed rest [4, 5]. Despite the fact that these patients were treated expertly with modern internal fixations such as intramedullary and extramedullary fixation, internal fixation related complications such as screw cut-out, nail removal, internal fixation breakage, vara deformity, and femoral neck shortening were reported to range from 6 to 21%, even as high as 30% in the elderly [6–9]. Blade cut-out, particularly the blade tip entering the hip joint without losing reduction (cut-through) [10], is still a common complication despite numerous incremental improvements. The dilemma is still an unsolved issue, and as the older population grows, its absolute numbers are probably going to rise as well. Several surgical salvage procedures, such as changing the helical blade, changing the blade with additional cement augmentation, reinserting the fracture nail, converting to hemiarthroplasty (HA), or total hip arthroplasty (THA), have been proposed to address these complications [10–12].

The recommended therapies for re-operation following unsuccessful intertrochanteric hip fracture repair are salvage osteosynthesis and hip arthroplasty [5, 13]. THA is frequently used as a salvage surgical procedure, but it is not ideal for these cut-through fractures due to the lack of loss of reduction. Re-nailing appears to be an effective rescue treatment.

**Fig. 1:**
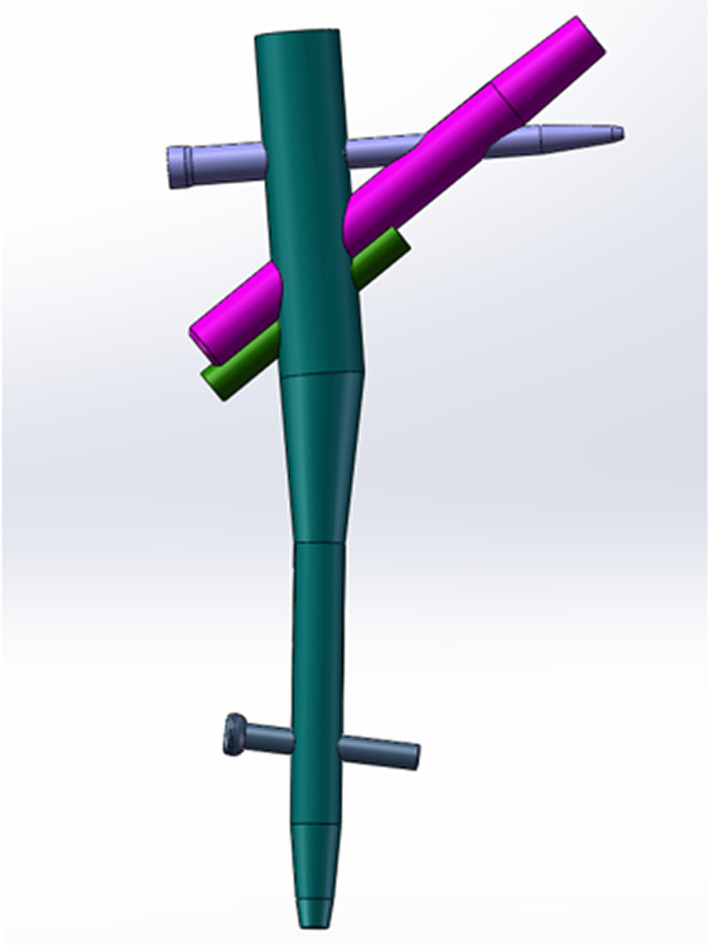
Three-dimensional model of the new type of proximal femur bionic intramedullary nail (PFBN)

However, no biomechanical studies on PFBN as a salvage treatment for intertrochanteric fractures with helical blade cut-through have been conducted to date. Thus, we explore the biomechanical stability of a new type of proximal femoral bionic intramedullary nail (PFBN) (Fig. 1) in the treatment of failed trochanteric fracture, which was designed by Professor Zhang et al. [14]. The proximal femoral bionic intramedullary nail (PFBN) is composed of the main intramedullary nail, compression screw, tension screw, additional compression screw, and distal screw (Fig. 1). The utilization of extra compression screws is determined by the specifics of the fracture. Therefore, we design a failed trochanteric fracture fixation model with spiral blade cut-through and compare PFBN with traditional intramedullary nails (PFNA) and dynamic hip screws (DHS) in the following re-operation by means of finite element analysis.

The purpose of this study is to compare the biomechanical properties of intramedullary fixation (PFBN and PFNA) versus extramedullary fixation (DHS) in the treatment of failed trochanteric fractures using finite element analysis. We hypothesized that PFBN would provide better biomechanical stability than PFNA and DHS due to its better anchoring structure.

## Methods

### Establish the cut-through fracture model and intramedullary and extramedullary internal fixation model

**Fig. 2:**
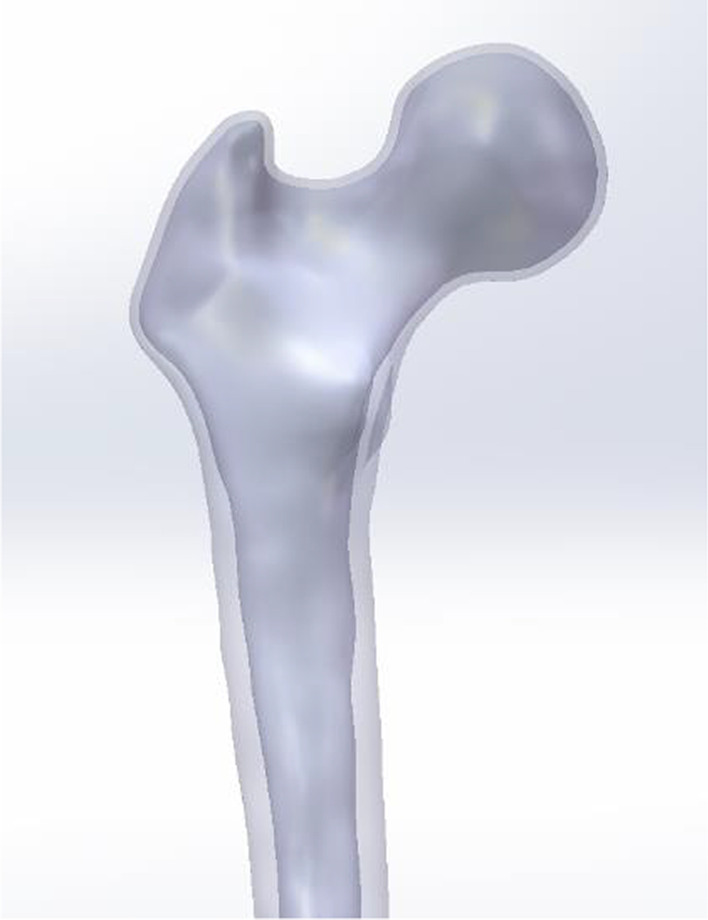
Three-dimensional model of the cortical bone and cancellous bone

**Fig. 3:**
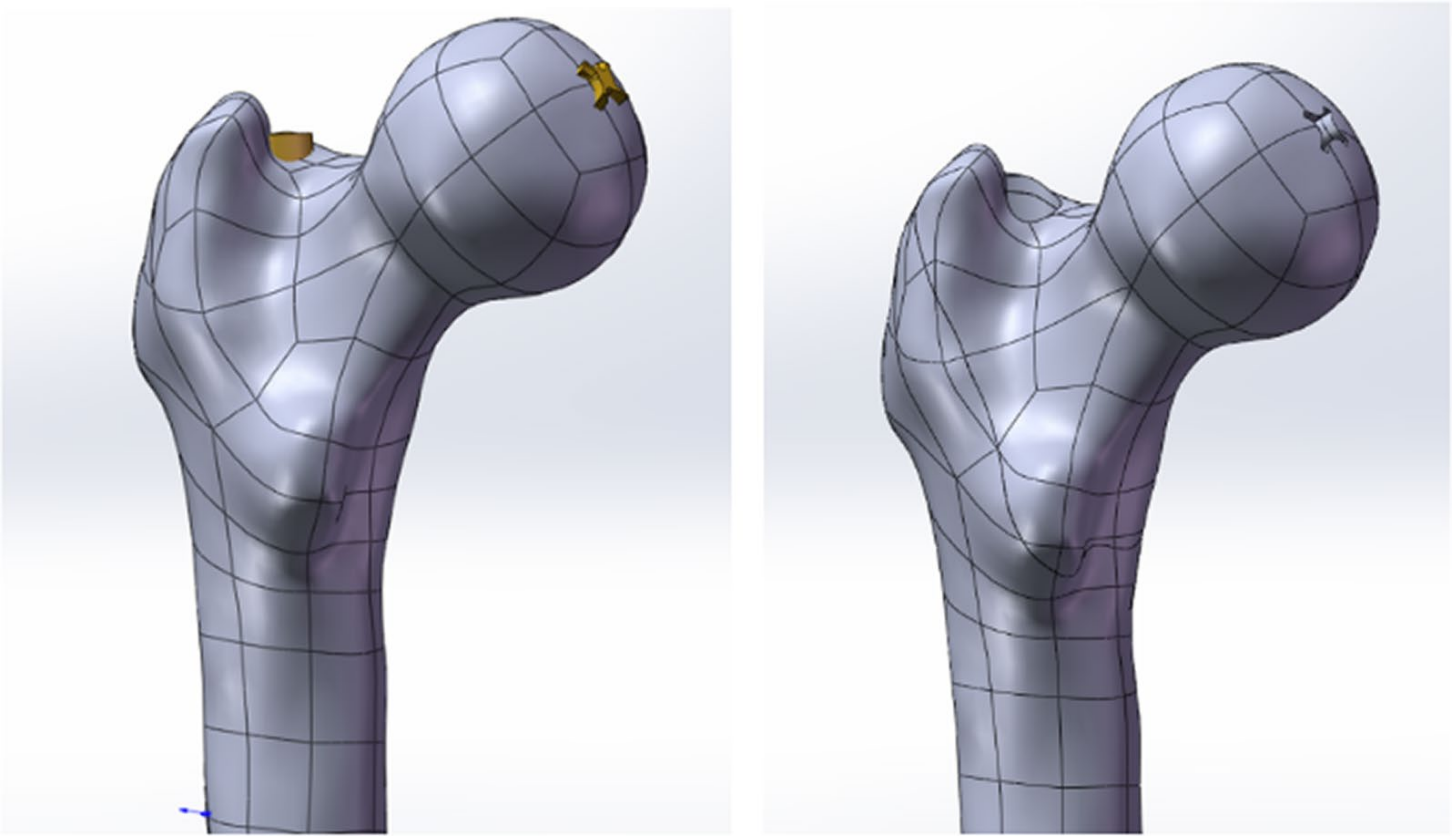
Three-dimensional trochanteric fracture cut-out model

**Fig. 4:**
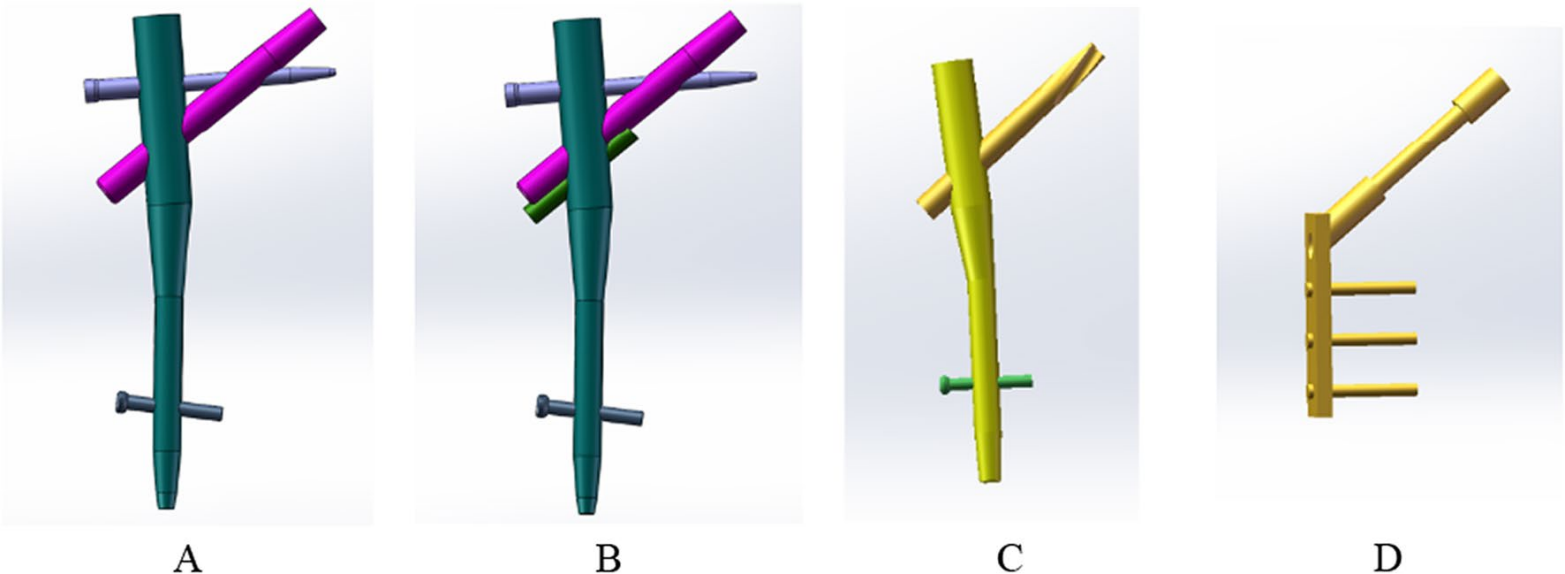
Internal Fixation Model: PFBN1 model (**A**), PFBN2 model (**B**), PFNA model (**C**), DHS model (**D**)

**Fig. 5:**
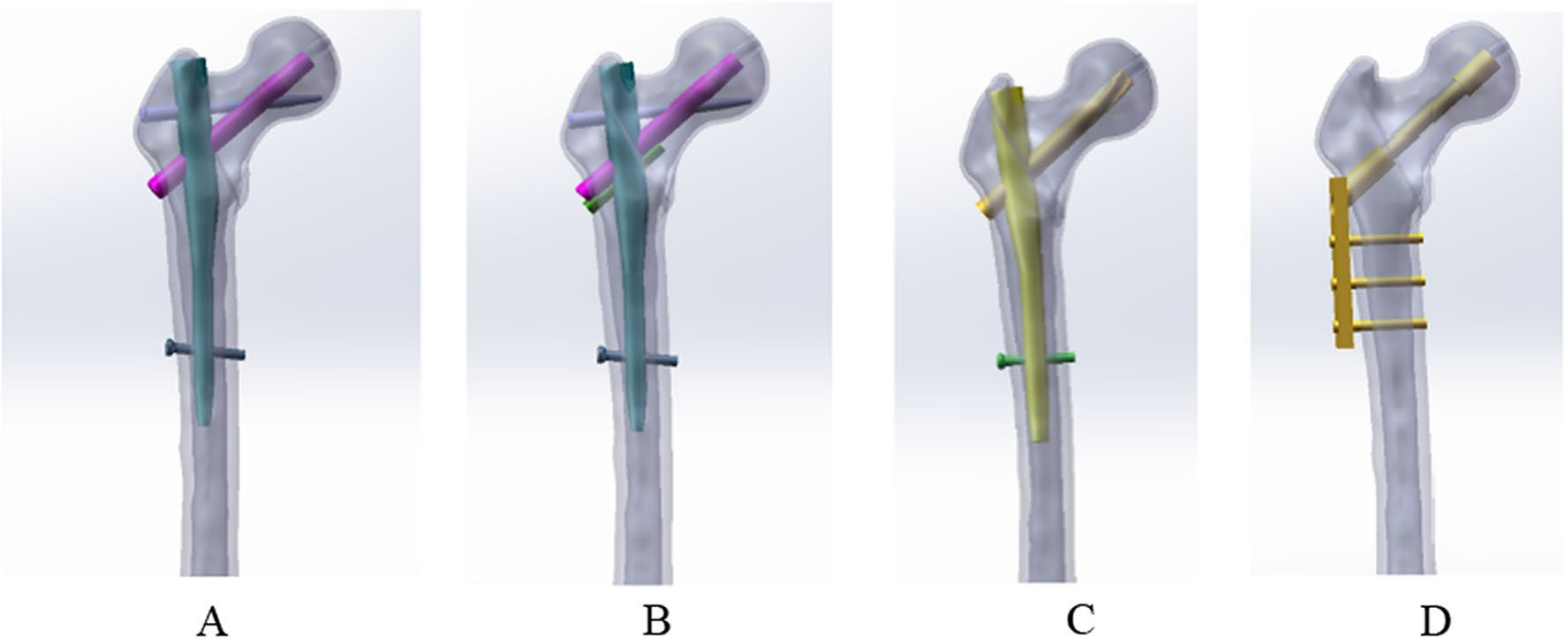
PFBN1 model (**A**), PFBN2 model (**B**), PFNA model (**C**), DHS model (**D**)

Using a Siemens 64-row CT scanner with a layer thickness of 0.7 mm, femur computed tomography (CT) data from a 26-year-old young male subject weighing roughly 70 kg were collected. The CT image has been stored in Digital Imaging and Communications in Medicine (DICOM) format and output to the Mimics 21.0 (Materialize, Leuven, Belgium) software for three-dimensional reconstruction to build a three-dimensional femoral model before being exported in STL format. These STL files were first imported into Geomagic Wrap 2017 (Geomagic, USA) software for smoothing, meshing, noise reduction, and surface fitting. Boolean procedures were used to create the three-dimensional models of the cortical and cancellous bones (Fig. 2), and a model of the proximal femoral bone was created for reassembly. They were then imported into SolidWorks 2017 (Dassault, France) software to determine the characteristics of the AO/OTA 31-A1.1 three-dimensional trochanteric fracture cut-thought model (Fig. 3). SolidWorks 2017 software (Dassault, France) was used to create the three-dimensional geometric model of the PFBN, PFNA, and DHS in accordance with the manufacturer's specified internal implant size (Fig. 4), finish assembling the intramedullary nail and extramedullary plate models (Fig. 5) and export the geometric model file. The difference between PFBN groups 1 and groups 2 is whether compression screws are implanted. Compression screws were not present in the PFBN1 group; however, they were present in the PFBN2 group (Fig. 5).

### Meshing

**Fig. 6:**
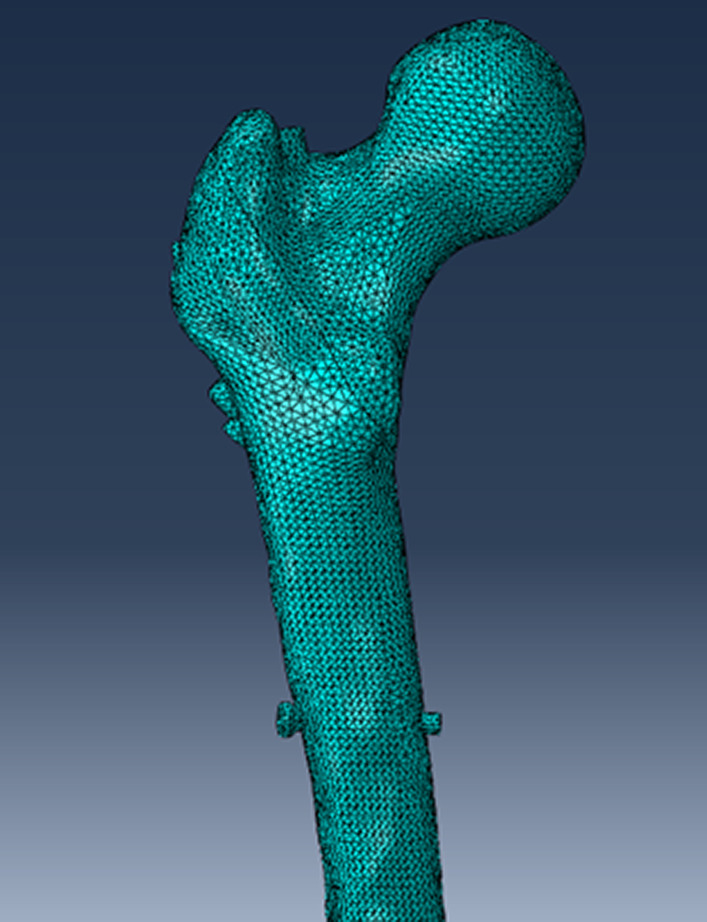
Meshing of PFBN2 group

The femur geometric fracture model and internal fixation were imported into the finite element analysis pre-processing software Abaqus 2017 (Simulia, France) for meshing (Fig. 6). The mesh size of the model is 1.5 mm, and the mesh quality has been checked and optimized. Each assembly was meshed by tetrahedral 10-node elements (C3D10). The number of nodes and elements in the four models is shown in Table 1.

**Table 1:** Details of the three assembly units and the total number of nodes

Case group	Node	Unit
PFBN1 group	493,206	318,722
PFBN2 group	413,542	262,543
PFNA group	474,291	304,883
DHS group	446,393	285,878

### Material parameters

All materials were assumed to be continuous, isotropic, and uniform linear elastic materials [15]. The elastic modulus of the bones and implants is listed in Table 2 with reference to the method recommended in the previous literature [9].

**Table 2:** Properties of the materials used in the present study

	Poisson’s ratio	*E* (GPa)
Titanium alloy	113,800	0.342
Cortical bone	17,000	0.3
Cancellous bone	445	0.2

### Model validation

To validate our finite element model, we reconstructed an intact femur model and set a vertical load of 1500 N applied to the femoral head according to a published experimental study [16]. Our results were similar to those obtained with previous experimental results [16], which means that the validity of this model has been verified, and can be used in future research.

### Boundary and loading conditions

**Fig. 7:**
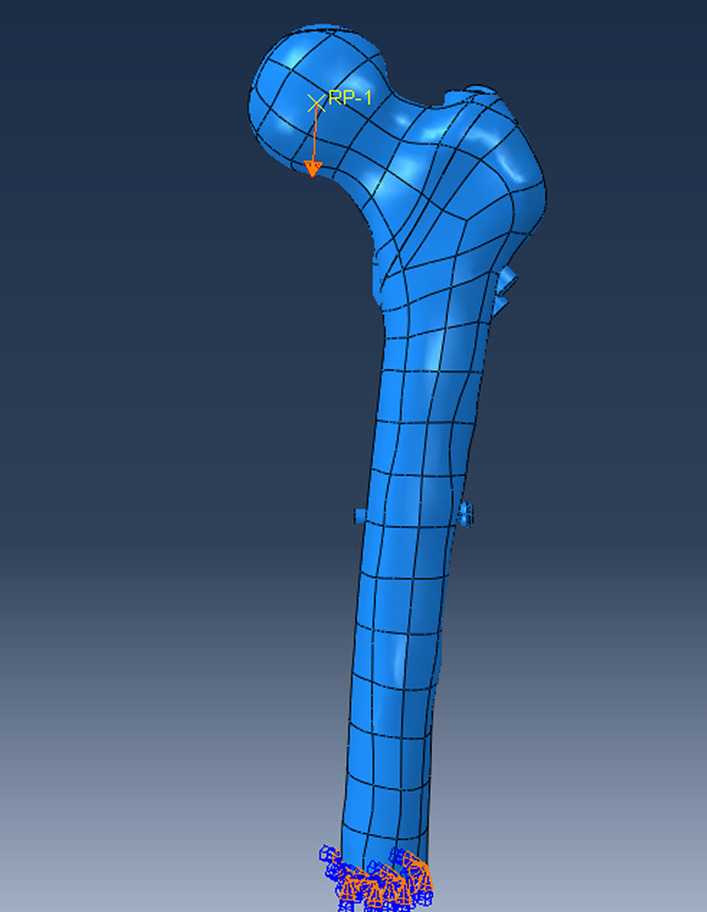
Loading and boundary conditions of PFBN2 model

In the finite element models, the load condition of 2100 N was applied to the center of the femoral head, the direction was normal standing angle vertical down, and the distal end of the femur was completely fixed (Fig. 7).

### Contact settings

The contact conditions were set as friction contact, the friction coefficient between bone and bone was 0.46 [17], the friction coefficient between bone–implant interactions was 0.3 [17], and the friction coefficient between implant–implant interactions was 0.2 [18].

### Evaluation criteria

The displacements and the von Mises stress distribution of the femur and internal fixations were measured in each group. The variation in each parameter was observed in each group.

## Results

### Model displacement of the femur

**Fig. 8:**
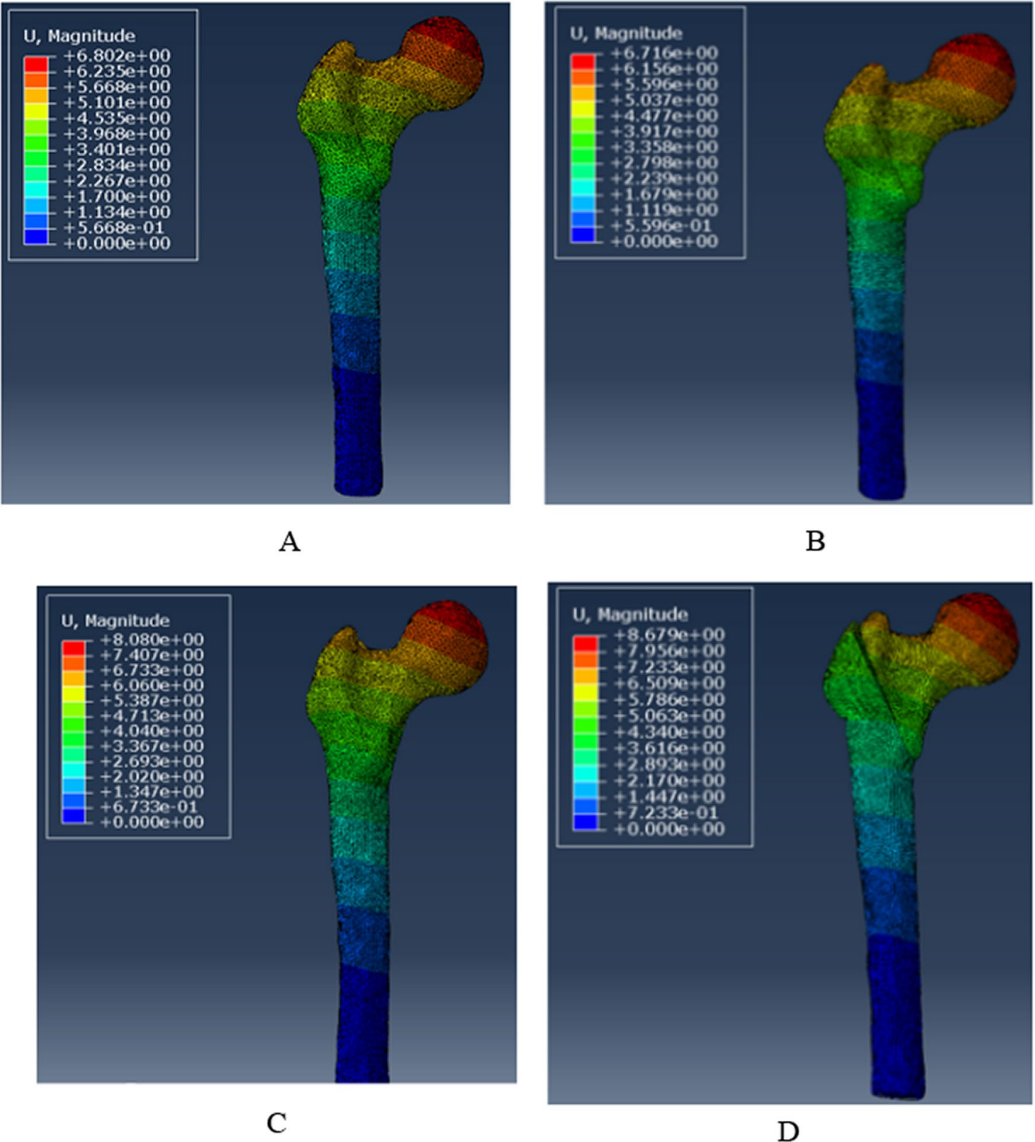
Maximum displacement of the femur: PFBN1 group (**A**), PFBN2 group (**B**), PFNA group (**C**), DHS group (**D**)

**Fig. 9:**
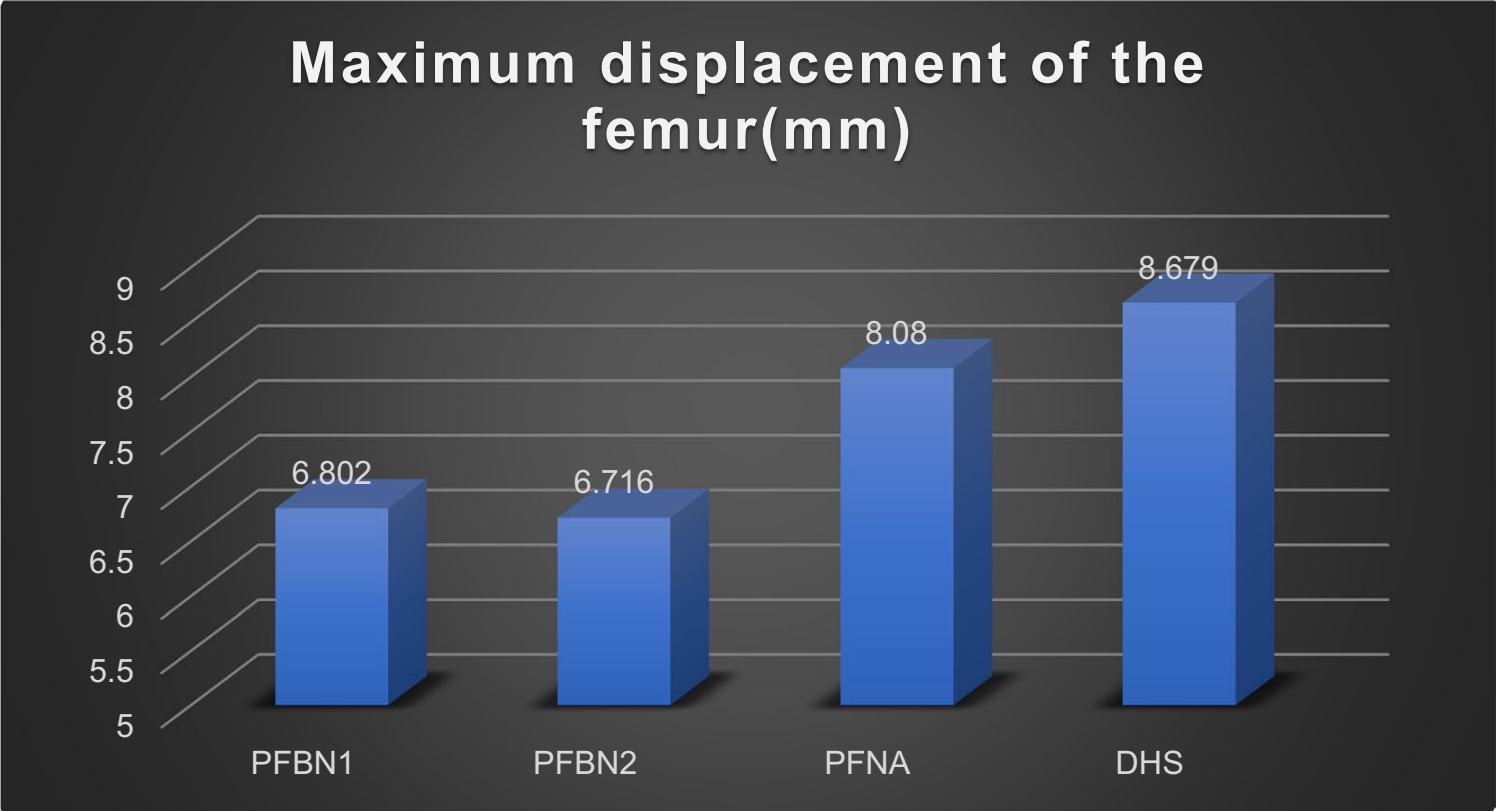
Graphic demonstration of the maximum displacement of the femur

Figure 8 depicts the deformation of the four groups of femoral models at the tip of the femoral head. The femoral displacement value in the PFBN1 group was 6.802 mm, 6.716 mm in the PFBN2 group, 8.080 mm in the PFNA group, and 8.679 mm in the DHS group, respectively (Fig. 9). The PFBN2 group had the least amount of femoral displacement. The PFBN1 and PFBN2 groups are nearly identical. While the DHS group experienced the greatest increase in displacement.

### Model displacement of the internal fixation

**Fig. 10:**
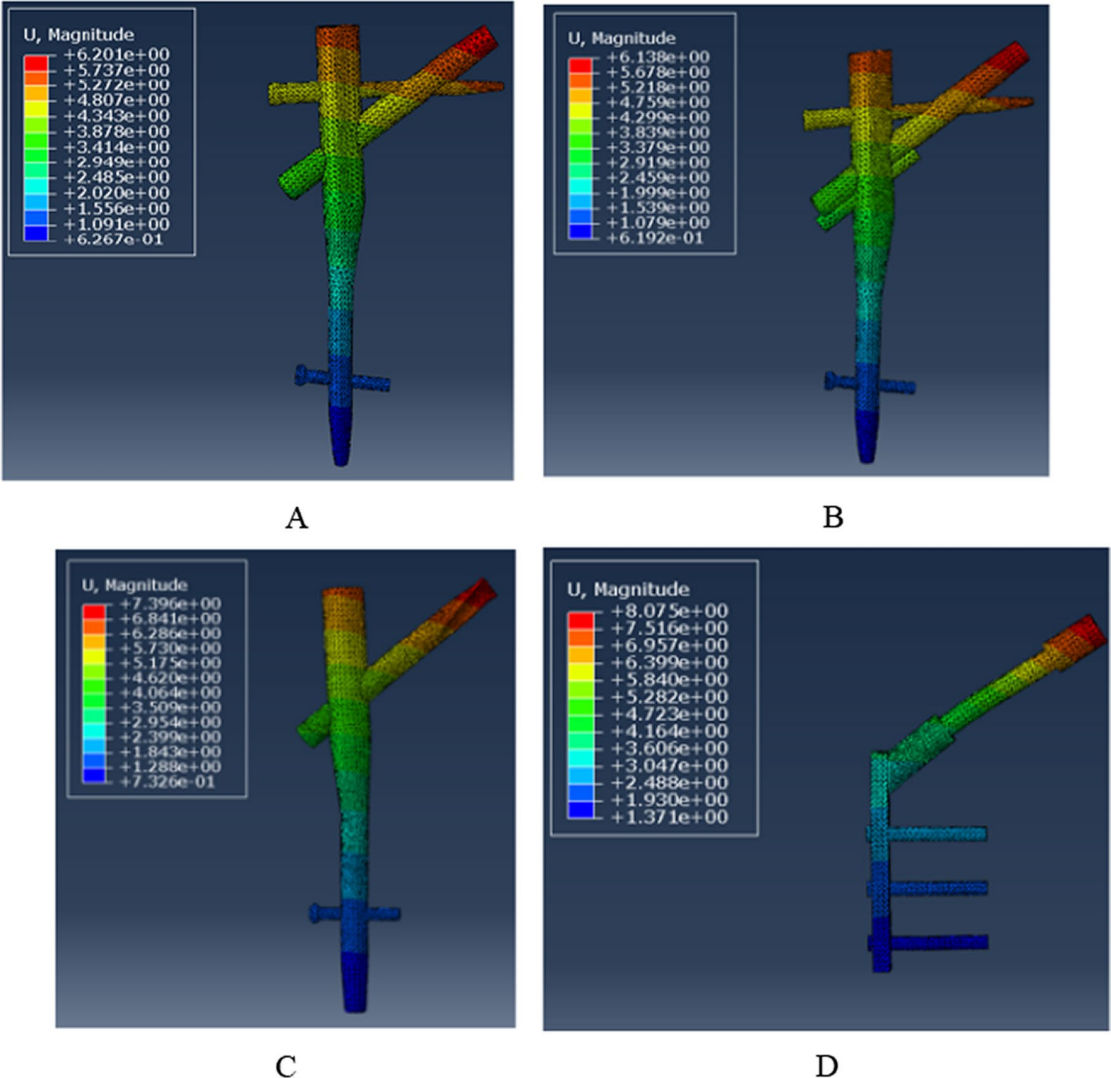
Maximum displacement of the internal fixation: PFBN1 group (**A**), PFBN2 group (**B**), PFNA group (**C**), DHS group (**D**)

**Fig. 11:**
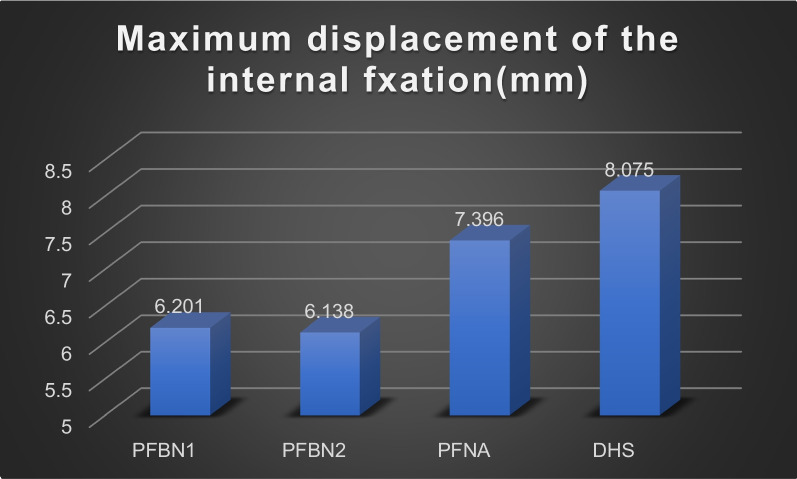
Graphic demonstration of the maximum displacement of internal fixation

As shown in Fig. 10, the deformation of the four internal fixation groups is concentrated at the screw's tip. Their respective displacement values were 6.201 mm for the PFBN1 group, 6.138 mm for the PFBN2 group, 7.396 mm for the PFNA group, and 8.075 mm for the DHS group (Fig. 11). The least displacement was in the PFBN2 group, followed by the PFBN1 group. The DHS group, however, was the worst.

### The von Mises stress of the femur

**Fig. 12:**
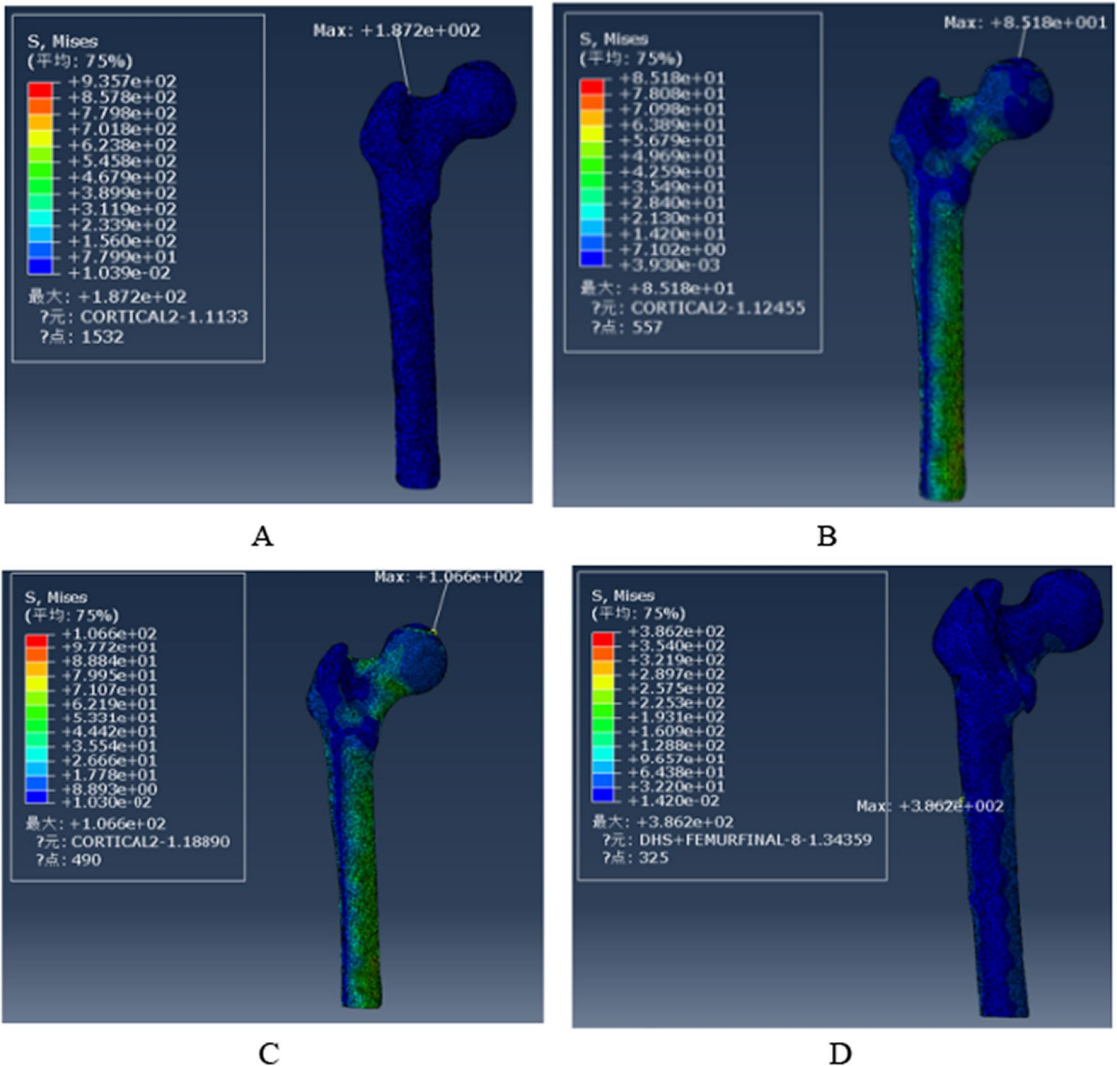
Maximum stress of the femur: PFBN1 group (**A**), PFBN2 group (**B**), PFNA group (**C**), DHS group (**D**)

**Fig. 13:**
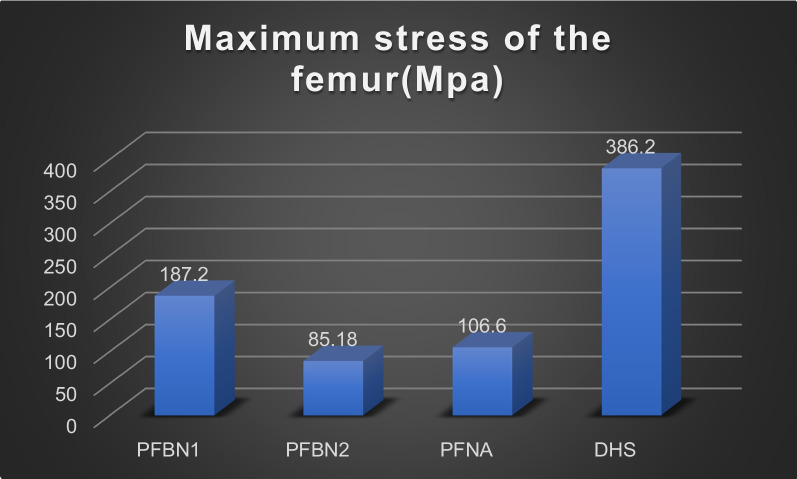
Graphic demonstration of the maximum stress of the femur

Von Mises Stress was displayed for four models of the femur in Fig. 12. The maximum stress in the PFBN1 group was 187.2 MPa at the intersection of the main nail and greater trochanter of the femur. The stress in the PFBN2 group was 85.18 MPa at the femoral head. The maximum stress in the PFNA group was 106.6 MPa at the femoral head. The maximum stress in the DHS group was 386.2 MPa at the intersection of the distal locking nail and the lateral cortex (Fig. 13). The PFBN2 group is the least stressed, followed by the PFNA group, the PFBN1 group, and the DHS group.

### The von Mises stress of the internal fixation

**Fig. 14:**
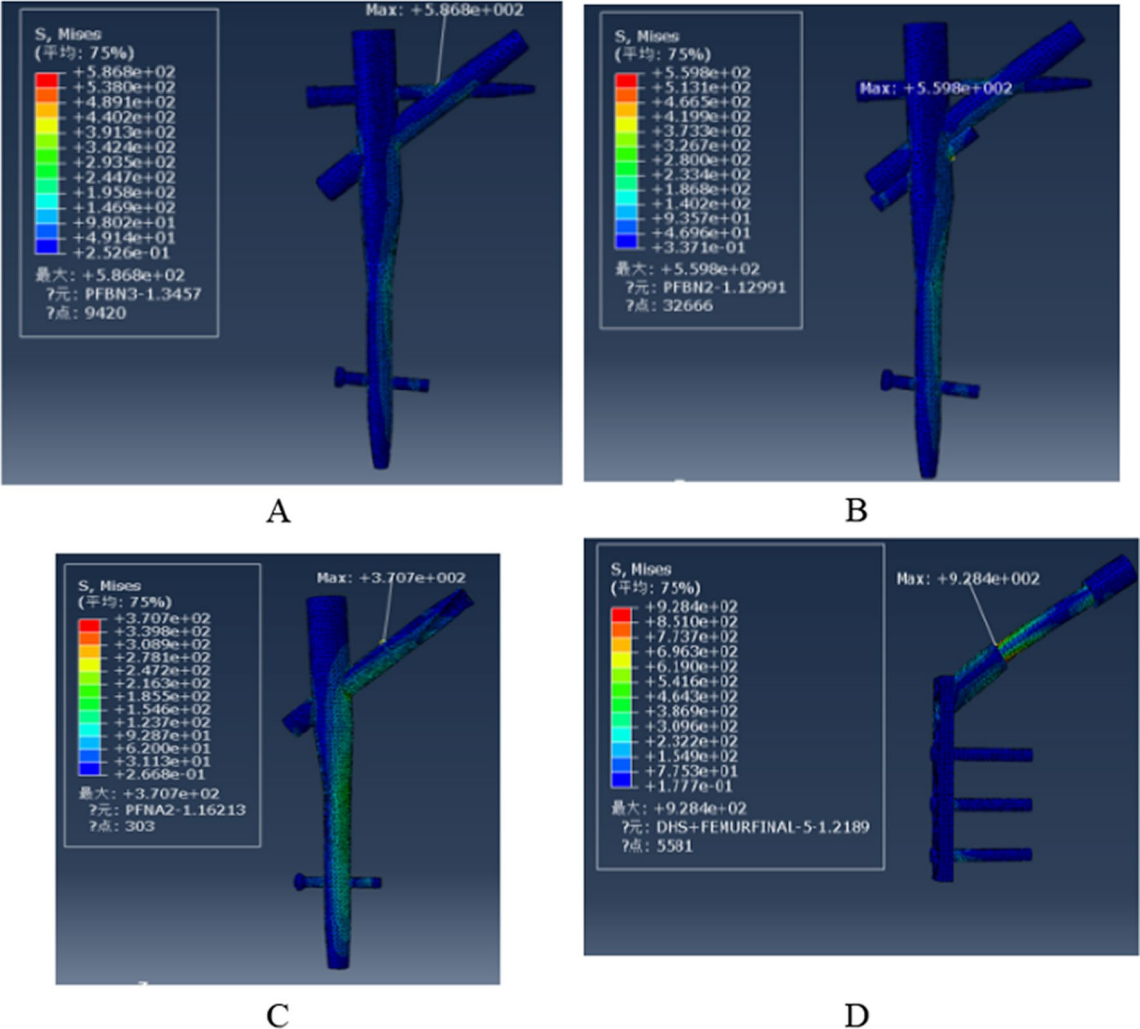
Maximum stress of FNS internal fixation: PFBN1 group (**A**), PFBN2 group (**B**), PFNA group (**C**), DHS group (**D**)

**Fig. 15:**
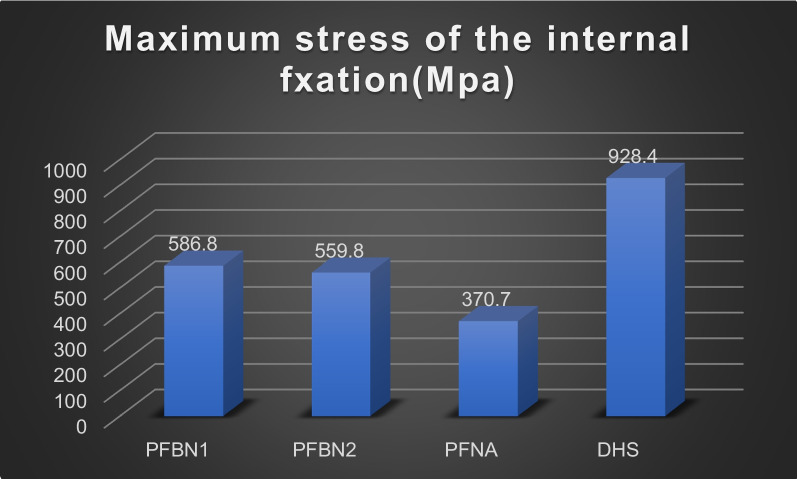
Graphic demonstration of the maximum displacement of internal fixation

Figure 14 shows the von Mises Stress distribution of four models of internal fixation. The maximum stress distribution of the PFBN1 groups was located at the junction of the pressure screw and tension screw. The maximum stress distribution of the PFBN2 groups was located at the junction of the main rod screw and pressure screw. The maximum stress distribution of the PFNA groups was located at the middle on the spiral blade. The maximum stress distribution of the DHS groups was located in the sleeve and of the sliding screw. The maximum von Mises Stress was 586.7 MPa, 559.8 MPa, 370.7 MPa, and 928.4.8 MPa in PFBN1 groups, PFBN2 groups, PFNA group, and DHS groups, respectively (Fig. 15). The PFNA group has the lowest stress, followed by the PFBN2 group, the PFBN1 group, and DHS group.

## Discussion

In our study, we explored the biomechanical outcome of a novel intramedullary (PFBN) and extramedullary approach to salvage treatment of failed internal fixations in intertrochanteric fracture. The least amount of femoral stress was found in the PFBN2 group, followed by the PFNA group, the PFBN1 group, and the DHS group. The DHS group, which was 4.53 times larger than the PFBN2 group, was noticeably larger. The same results were found for the internal fixation stress: the intramedullary nail system had the smallest stress, but the stress was significantly higher in the DHS group, which was 2.15 times that of the PFNA group.

In terms of femoral and internal fixation stability, the smallest femoral and internal fixation displacement was observed in the PFBN2 group, which was followed by the PFBN1 group, the PFNA group, and the DHS group. When compared to intramedullary fixation, the displacement of the femur and internal fixation in the DHS group increased by 1.39 times. This indicates that in the second operation for the femoral trochanter fracture, the DHS group is less stable than the intramedullary nail. This could be due to the following factors: On the one hand, it is a stable intertrochanteric fracture, and the fracture type is AO/OTA 31-A1.1. Because PFBN can be implanted with additional compression screws below the compression screws, the fixing effect of PFBN on femoral calcar separation and displacement may be improved. On the other hand, the lever-balance reconstruction of PFBN also has advantages in stability anchoring over PFNA. Therefore, the result of PFBN groups is more stable than that of PFNA groups. While PFNA can generate bone compression around the helical blade because there are bone deficiencies in the femoral neck and femoral head after PFNA revision, which is more beneficial than DHS. This contradicts the findings of Baca et al. [19]. They investigated the location of intramedullary hip screws for implantation in stabilized trochanteric fractures. According to their findings, displacement is greater in unstable intertrochanteric fractures with bone defects. On the other hand, in stable fractures, outcomes do not require absolute precision, and minor deflections in the placements of the nails and neck screws do not considerably raise the risk of failure for the entire fixation [19]. The primary reason for the divergence is that we compare several types of intramedullary and extramedullary nails, whereas the latter compares the position of a single intramedullary nail. The displacement differences between the PFBN1 and PFBN2 groups are negligible when using the same intramedullary nail type as in our study. Our results in the same intramedullary nail type comparison were comparable to the latter's. In revision surgery of intertrochanteric fractures, extramedullary fixation, such as DHS internal fixation, is not recommended.

Intertrochanteric fractures have been associated with two main forms of implant-related complications, known as cut-out and cut-through. Cut-out refers to the perforation of the helical blade through the superior cortex of the femoral head or neck, followed by rotation and varus collapse of the head-neck fragment; and cut-through refers to the blade's medial migration, with perforation of the blade tip into the hip joint without loss of reduction [10]. There is a common complication of extramedullary and intramedullary implants, and it is primarily caused by excessive sliding of the screws and helical blade, or femoral medialization, which is the major cause of implant failure in the fixation of trochanteric fractures. Simmermacher et al. [20] conducted a multicenter clinical study and found that there was a 2.3% cut-out rate with the PFNA. Another study reported that the PFNA blade cutout rate was 3.6% for the treatment of unstable proximal femoral fractures [21]. However, the effective treatment for intertrochanteric fracture after internal fixation failure remains uncertain. The different studies published so far do not seem to agree on the optimal treatment for revision of intertrochanteric fractures.

The lever-balance reconstruction hypothesis of internal fixation for treating intertrochanteric fractures was proposed by Zhang et al. [14] in response to complications associated with internal fixation. According to the theory, the normal structure of the proximal femur is similar to a lever, with the fulcrum near the center of the femoral head. The lateral tension arm of the femur is longer than the medial pressure arm, which allows it to withstand more compressive stress. The original lever system was destroyed following the fracture. The goal of internal fixation is to replace the original lever system with a new lever system that is closer to the original anatomical fulcrum. A novel kind of proximal femoral bionic intramedullary nail (PFBN), which combines a main nail, a pressure screw, and a tension screw to create a stable structure, was developed based on the theory. The utilization of additional compression screws is determined by the specifics of the fracture. Theoretically, it has better stability because it can withstand the compressive and tensile stresses brought on by weight-bearing after the fracture operation.

In actuality, properly selected patients can achieve excellent success rates for revision surgery [22, 23]. Determine salvage alternatives based on the patient's age, gender, daily function, functional needs, life expectancy, fracture comminution grade, remaining bone mass, and fragility [24, 25]. Said et al. [26] reported a study of failed DHS fixations of intertrochanteric fractures and found that patients in the revision internal fixation can achieve fracture union without bone grafting at a mean time of 17 weeks. A favorable functional outcome following revision internal fixation was found in retrospective research [27], and the Barthel index and SF-12 were used to measure quality of life. According to studies done by Tucker et al. [28] following the failure of a cephalomedullary nail (CMN), revision intramedullary nailing has a faster rate of fracture union than plate treatment and a lower mortality rate (25%) than both joint replacement and plate treatment (33%) combined. This can be explained by the fact that intramedullary fixation is clearly superior to extramedullary fixation in terms of internal fixation stability, which is consistent with our research. They have tiny lever arms and bending loads that are up to 30% lower than those of extramedullary devices.

Albareda et al. [29] found that the hip preservation treatment of a variable angle femoral plate is an attractive option with good outcomes and few complications in the cut-out treatment. A multicenter study by Brunner et al. [10] compared a number of treatments, from solitary lag screw exchange to THA, and came to the conclusion that THA was the best option with the fewest problems. Sebastián et al. [30] reported that hip replacement appeared to be a relatively safe and reliable salvage procedure for elderly and physically frail patients. Tetsunaga et al.^31^ report that the rate of postoperative complications was significantly higher in the group of patients who had trochanteric fractures than in the group who had femoral neck fractures (25 vs. 0%, *p* 0.0001), which raises questions about the superiority of hip replacement in all patients who had this complication. In cases of intertrochanteric fractures where internal fixation has failed, hip replacement is a challenging and intricate procedure.

In summary, hip replacement is more appropriate for patients with low bone quality and insufficient bone mass in the elderly population, and salvage osteosynthesis is preferable for individuals who are young, have a long-life expectancy, and have sufficient bone quality for fixation. Our findings suggest that PFBN is one of the most reliable internal options for intertrochanteric fracture revision. Compared with PFNA and DHS, the postoperative displacement of the femoral end and internal fixation is smaller than the former, showing better biomechanical stability. This is explained by the following factors: To begin, PFBN differs from PFNA in that it has one tension screw in the greater trochanter and one additional compression screw in the lesser trochanter. This triangle stabilizing technique can greatly improve internal fixation stability and reduce the possibility of screws cutting out. Second, unlike the eccentric fixation of DHS, PFBN has a central fixation with a more balanced biomechanical distribution. As a result, we advocate intramedullary fixation for salvage osteosynthesis since it is more stable and less intrusive than extramedullary fixation.

### Limitation and strength

There are some limitations to this study. First, as in other finite element studies, the FEA model in this study is set as a homogeneous, continuous, and isotropic elastic material. Because human bone is an isotropic heterogeneous material, the material properties in the finite element experiment may have an impact on the final results. Second, this study only performed static mechanical analysis and did not include dynamic mechanical analysis. Human body activity is a compound, dynamic process that will require more dynamic mechanical analysis in the future. Notwithstanding these limitations, to the best of the authors' knowledge, this was the first study to use finite element analysis to examine the biomechanical effectiveness of intramedullary and extramedullary treatment for internal fixation failure of intertrochanteric fractures. Cadaveric biomechanical studies and randomized clinical trials are still advocated to support these findings.

## Conclusion

Our biomechanical research demonstrates that intramedullary fixation is more stable than extramedullary fixation when salvaging failed internal fixations in intertrochanteric fracture. Compared with PFNA and DHS, PFBN showed better biomechanical stability in the treatment of patients with revised intertrochanteric fractures. In light of this, we advocate PFBN fixation as the method of choice for intertrochanteric fracture revision. This result still has to be confirmed in more clinical research.

## Data Availability

The datasets used and analyzed during the current study are available from the corresponding author on reasonable request.
